# Type I error rates and power of two randomization test procedures for the changing criterion design

**DOI:** 10.3758/s13428-023-02303-1

**Published:** 2023-12-11

**Authors:** Rumen Manolov, René Tanious

**Affiliations:** 1 Department of Social Psychology and Quantitative Psychology, Faculty of Psychology, University of Barcelona, Passeig de la Vall d’Hebron 171, 08035 Barcelona, Spain; 2 Faculty of Psychology and Neuroscience, Maastricht University, Universiteitssingel 40, 6229 ER Maastricht, the Netherlands

**Keywords:** Changing criterion design, Randomization test, Power, Simulation study

## Abstract

Single-case experimental design (SCED) data can be analyzed following different approaches. One of the first historically proposed options is randomizations tests, benefiting from the inclusion of randomization in the design: a desirable methodological feature. Randomization tests have become more feasible with the availability of computational resources, and such tests have been proposed for all major types of SCEDs: multiple-baseline, reversal/withdrawal, alternating treatments, and changing criterion designs. The focus of the current text is on the last of these, given that they have not been the subject of any previous simulation study. Specifically, we estimate type I error rates and statistical power for two different randomization procedures applicable to changing criterion designs: the phase change moment randomization and the blocked alternating criterion randomization. We include different series lengths, number of phases, levels of autocorrelation, and random variability. The results suggest that type I error rates are generally controlled and that sufficient power can be achieved with as few as 28–30 measurements for independent data, although more measurements are needed in case of positive autocorrelation. The presence of a reversal to a previous criterion level is beneficial. R code is provided for carrying out randomization tests following the two randomization procedures.

The changing criterion design (CCD) is one of the designs available to researchers planning to conduct a single-case experiment^1^. The CCD has a long-standing tradition in single-case experimental design (SCED) research since its formal introduction by Hartman & Hall (1976) half a century ago. The design is particularly useful with behavior “where an immediate, considerable increase or decrease may be difficult to achieve or undesired; therefore, gradual shifts toward a desired goal are applied” (Klein et al., 2017, p. 52). In practical terms, this means that after initial baseline measurements, the researcher determines—oftentimes in consultation with the participant(s)—a criterion that the participant has to meet in the subsequent experimental phase when the treatment is administered (e.g., drinking five alcoholic beverages with a finacial incentive in place). This criterion is either increased or decreased during the course of the study, depending on the nature of the behavior in question, but it is recommended to incorporate mini-reversals to a previous criterion to increase internal validity (Klein et al., 2017). Due to this stepwise nature of the CCD and the fact that the intervention is usually not withdrawn after the initial baseline period, appropriate randomization procedures have long been lacking for this design relative to other major SCEDs (i.e., phase designs [with an initial proposal by Onghena, 1992], multiple baseline designs [see Levin et al., 2018, for a review of alternatives], and alternation designs [e.g., Onghena & Edgington, 1994; Manolov, 2019]). Systematic reviews have repeatedly indicated that the CCD as described here is less frequently used than these other design options (Shadish & Sullivan, 2011; Tanious & Onghena, 2021; Smith, 2012) in spite of the great value of the design.

## Two randomization procedures for the changing criterion design

Randomization has been highlighted as a means of increasing internal validity (Kratochwill & Levin, 2010) and has been recently emphasized again when considering the need to upgrade design standards (Kratochwill et al., 2023). Specifically, Kratochwill et al. (2023) recommend being explicit about the randomization procedure used. However, when referring to randomization in the design and the possibility to use a randomization test, they only comment on multiple-baseline, reversal, and alternating treatments designs. CCDs are also missing from their previous commentary on the What Works Clearinghouse standards (Kratochwill et al., 2021), but we consider that these designs can also benefit from using randomization in the design and having an additional option for data analysis (i.e., randomization tests). Randomization procedures have long been unavailable for this design, presumably calling into question the scientific credibility of the design (Kratochwill & Levin, 2014). Randomization procedures specifically for the CCD have only recently been proposed. In general, randomization procedures for single-case experiments refer to the random assignment of treatments to treatment times (Edgington, 1980a, 1987, 1996). For the CCD, however, where only one treatment is administered and baseline measurements are usually taken only at the beginning of the study, that general way of randomizing does not work. In recent years, three randomization procedures have been proposed to fill this gap: the phase change moment randomization (Ferron et al., 2023; Onghena et al., 2019) related to the proposal by Onghena (1992) for reversal designs, the blocked alternating criterion randomization (Tanious, 2022), and the completely randomized alternating criterion procedure (Tanious, 2022). The first two procedures preserve the stepwise nature of the CCD and are therefore the subject of the present simulation study. The third randomization procedure does not follow the stepwise nature and should therefore be investigated separately. In the following sections, both randomization procedures will first be explained and then demonstrated with real-life data.

### Phase change moment randomization

**Fig. 1:**
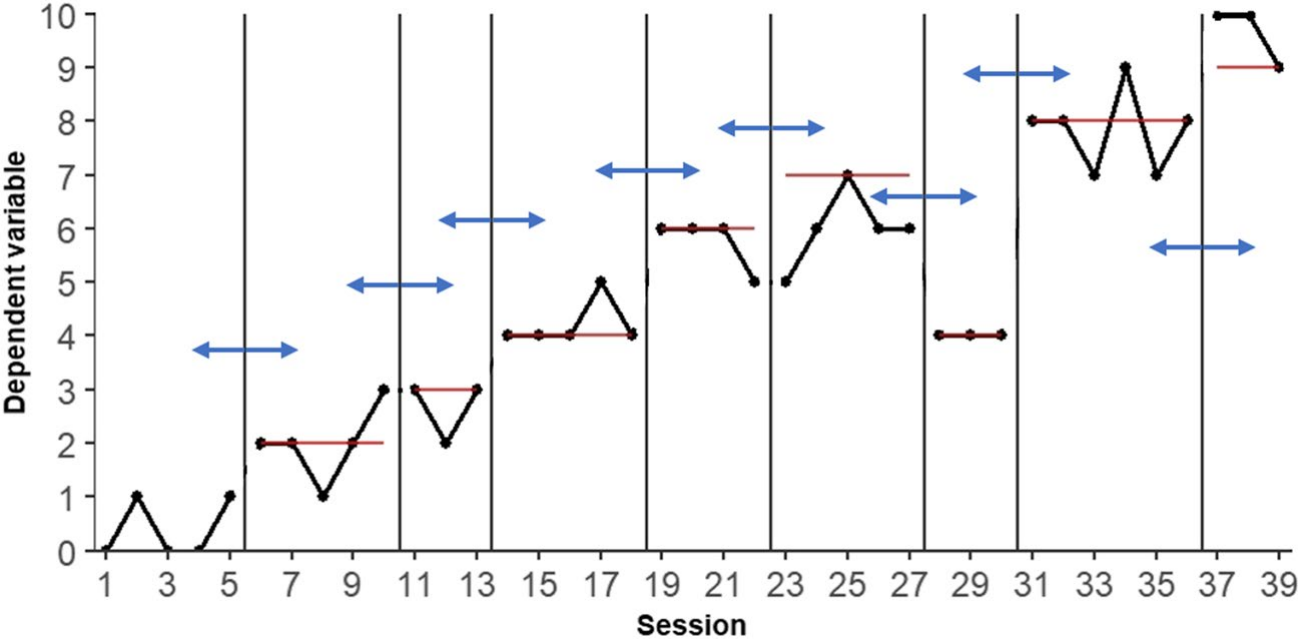
Hypothetical data illustrating the phase change moment randomization procedure. *Note.* The first phase is the baseline, which does not include any criterion level. The criterion levels for the intervention phases are marked with a red horizontal line. The blue horizontal arrows mark the transitions between phases

Phase change moment randomization (PCM; Onghena et al., 2019) relies on randomly determining the moment of criterion change between adjacent phases. Figure 1 demonstrates this procedure with hypothetical data.

The data depicted in Fig. 1 consist of nine phases: a baseline phase with no criterion in place and eight experimental phases with different criteria to be met by the participant. With nine total phases, there are eight phase change moments that can occur at random moments under certain constraints which the researcher determines a priori. As Onghena et al. (2019) put it, “the random assignment procedure for the changing criterion design involves defining a population of potential phase change points and randomly selecting the phase change points for the actual experiment” (p. 22). The number of permissible randomizations under this procedure depends on a number of a priori decisions, such as the total number of measurements and the minimal number of measurements per phase.

### Blocked alternating criterion randomization

**Fig. 2:**
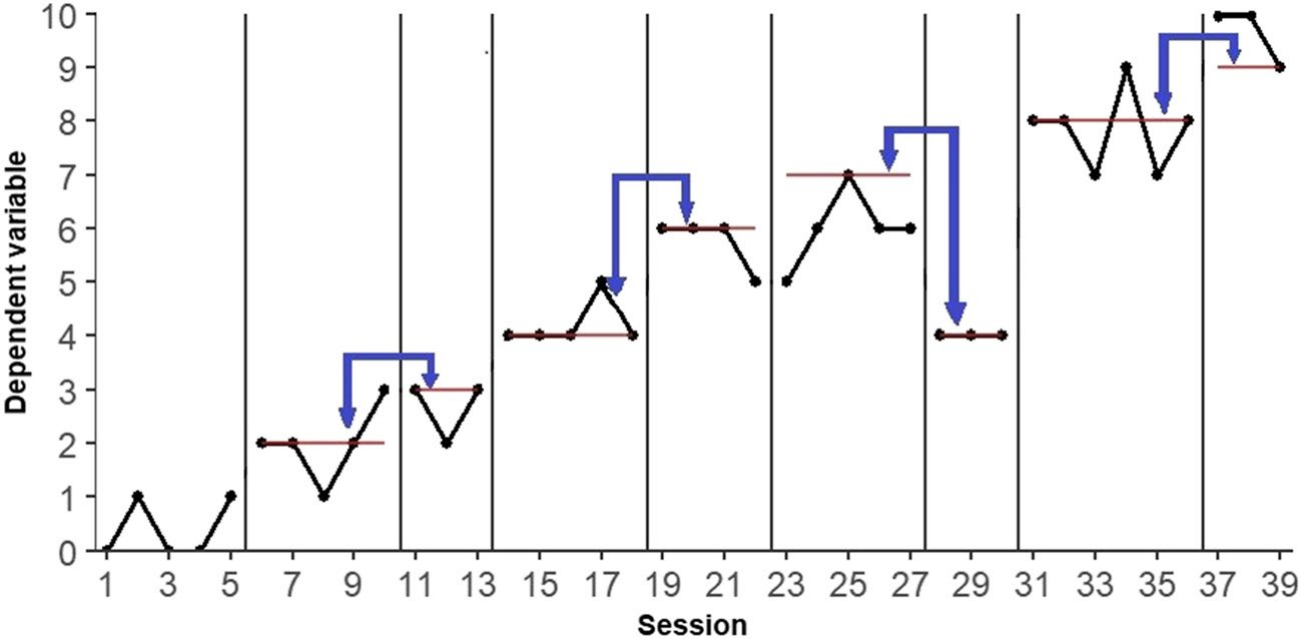
Hypothetical data illustrating the blocked alternating criterion randomization *procedure*. *Note.* The first phase is the baseline, which does not include any criterion level. The criterion levels for the intervention phases are marked with a red horizontal line. The blue arrows mark the possible orders between successive blocked pairs of phases

Blocked alternating criterion randomization (BAC; Tanious, 2022) relies on the random determination of the order in which two adjacent criteria are presented. This procedure incorporates mini-reversals by default. Figure 2 demonstrates this procedure with the same hypothetical data as for PCM.

The criteria can be segmented into blocks in a similar way as originally proposed by Edgington (1980a) for alternating treatments designs. Within each block, the order of criterion implementation can be determined randomly. The stepwise nature of the changing criterion design is best preserved when segmenting the criteria into blocks of two (Tanious, 2022). When segmenting the criteria into blocks of two, the number of possible randomizations with the BAC procedure equals two to the power of the number of blocks. Thus, for the data depicted in Fig. 2, the number of randomizations is ${2}^{4}=16$. The main a priori decision on which the number of randomizations with BAC depends is the number of criteria, which partly depends on the number of total measurements. Finally, it should be noted that in order to segment all criteria into blocks of two, an even number of criteria is needed. With an uneven number of criteria, the last or any other block can be segmented into three criteria, but this is not included in the current simulation study.

## Similarities and key differences between the procedures

### Similarities

Before moving on to an example for both procedures and a discussion of the simulation conditions, it is important to highlight the similarities and key differences between the two procedures. The generic steps for executing both procedures are the same: choose the design (in this case, CCD), formulate hypotheses, determine the number of measurements and set the nominal significance level (alpha), choose a test statistic sensitive to the desired effect (in this case, the mean absolute deviation [MAD], as suggested by Onghena et al., 2019), determine the randomization procedure (here, PCM or BAC), choose one of the admissible randomizations and conduct the experiment, calculate the observed test statistic, construct the randomization distribution, and determine the *p*-value (Bulté & Onghena, 2008; Edgington, 1967, 1975; Tanious & Manolov, 2023). Within this generic framework, the two procedures are situated in the step “determine the randomization procedure,” the choice of which has implications for the subsequent steps “choose one of the possible randomizations,” “construct the randomization distribution,” and “determine the *p*-value.”

### Conceptual differences

The most notable conceptual difference between the two procedures is that PCM randomly determines the moment at which the change from one criterion to the following occurs, whereas BAC randomly determines which criterion is implemented between adjacent phases. In practice, this means that with a strict application of PCM, the phase lengths are determined by the randomization procedure, even though Onghena et al. (2019) refer to the procedures described in Edgington (1980b) for response-guided randomization procedures. Thus, on the one hand, BAC offers the advantage of having flexible phase lengths, which may be advantageous if the researchers consider it important to reach stability in each phase (i.e., for each criterion) before moving on to the next one (Hartmann & Hall, 1976; Klein et al., 2017). On the other hand, PCM lets the researchers determine a priori the order in which the criteria are presented and subsequently randomizes the moment at which the criterion changes occur. When following the BAC procedure, the order of criterion implementation is determined by the randomization procedure in a restricted manner determined by the researchers a priori, but the phase lengths can be adapted to the practical circumstances. Both features of the two procedures have important implications in the context of CCDs. CCDs rely on the stepwise increase or decrease in target behavior. With the PCM procedure, researchers can plan in advance the exact manner of this stepwise implementation and the extent of criterion increase or decrease between successive phases. It could, however, happen that the randomization procedure proscribes a criterion change before the participant reaches the criterion in the current phase for a specified number of successive measurements. With the BAC procedure, the researchers have less flexibility in determining the exact degree of criterion increase or decrease between successive phases. The criteria will increase or decrease over the course of the study to the desired level, but BAC includes mini-reversals by default, which may not always be feasible in practical or ethical terms.

A second conceptual difference is related to the fact that statistical power depends on the number of possible randomizations, and for PCM and BAC this number depends on different factors. Specifically, with PCM, the number of possible randomizations depends mainly—but not exclusively—on the number of measurements, both the overall number of measurements and the possible criterion change moments related to the minimal desired phase length (Michiels & Onghena, 2019; Onghena, 1992). This allows for obtaining reasonable power in experiments with fewer phases if the overall number of measurements is sufficiently high. On the contrary, with BAC, the number of possible randomizations depends on the number of phases and how they are divided into blocks. This allows for obtaining reasonable power even with shorter phases if the number of phases is sufficiently high.

A third conceptual difference is related to how the baseline data are treated and how the baseline is incorporated into the randomization procedure. For BAC, the baseline phase is not included in the randomization procedure because there is no criterion present in the baseline phase that could be randomized. For PCM, relating to the use of the baseline data when computing the test statistic, Onghena et al. (2019); Tanious (2022) suggest comparing the baseline measurements to their median. Not discarding the baseline data also entails that there is an additional moment of change in phase (from the baseline to the first intervention phase condition), which is related to having more possible randomizations. Both including the baseline data and having more randomizations is likely to be related to greater statistical power. However, here we argue that it is preferable not to include the baseline in the randomization procedure and test statistic calculation for two reasons. First, it has repeatedly been recommended to wait for baseline stability before introducing the intervention (Ferron et al., 2017; Kazdin, 2019; Krasny-Pacini, 2023; Ledford et al., 2019), which requires flexible baseline length. Second, when it comes to calculating the observed test statistic, there is no criterion present in the baseline phase that the scores can be compared to. Onghena et al. (2019) suggest using the median baseline value for that purpose, but that criterion was not present when actually collecting the data. Thus, to avoid comparing the data to an arbitrary criterion and to have flexible baseline length, we consider it preferable to not include the baseline in the randomization test procedures. In the current text, the illustration provided later follows the idea of excluding the baseline data, and thus the same data are used for both BAC and PCM.

### Computational differences

Apart from these conceptual differences, some a priori computational differences between the two procedures are noteworthy. These computational differences play a role in setting meaningful simulation conditions. The number of possible randomizations under the two randomization procedures is very different, for the same series length and the same number of phases. For instance, for $n=20$ and four phases, there would be 165 randomizations under PCM if the minimal phase length is 3 (and the maximal is 11), whereas there would only be four randomizations under BAC (with the four phases organized into two blocks and there being five measurements per phase, although the number of measurements per phase does not have an influence on the number of randomizations for BAC). Similarly, for $n=40$ and eight phases, there would be 245,157 randomizations under PCM if the minimal phase length is 3 (and the maximal is 19), whereas there would only be 16 randomizations under BAC (with the eight phases organized into four blocks of two and there being five measurements per phase). Consequently, for detecting an effect at $\alpha =.05$, BAC requires at least $n=30$ (if each phase should contain at least three measurements) and ten experimental phases (i.e., five blocks), leading to 32 randomizations. For this series length and this number of phases, there would be 1716 randomizations under PCM if the minimal phase length is 3 (and the maximal is 9). Conversely, for detecting an effect at $\alpha =.05$, PCM only requires $n=15$ and four phases, if the minimal phase length is 3 (and the maximal is 6), leading to 20 randomizations.

However, under PCM (but not under BAC), listing systematically all combinations becomes computationally very difficult when the number of phases and/or the number of possible phase lengths increases. At the same time, selecting random samples of all possible PCM combinations is also not computationally easy, because these random samples should all include phases with lengths between the minimal and the maximal value while leading to the same total series length. This is computationally challenging because the length of the initial phase(s) influences the lengths of subsequent phase(s). For instance, for $n=15$ and four phases, with a minimal phase length of 3 (and maximal of 6), each phase can theoretically have one of four possible lengths: 3, 4, 5, and 6. This means that there are ${4}^{4} = 256$ possible combinations of these four lengths for four phases. However, out of these 256 combinations, only 165 lead to a series length of 15 (i.e., there are 165 randomizations in this case), as per the formula by Onghena (1992), which will be shown by example in the next paragraph. For example, if all four phases have a length of 3, the total length will be 12, whereas if all four phases have a length of 6, the total will be 24, none of which leads to a series length of 15. Similarly, for $n=19$ and five phases, if the minimal phase length is 3 (and the maximal is 7), each phase can theoretically have one of five possible lengths: 3, 4, 5, 6, and 7. This means that there are ${5}^{5} = 3125$ possible combinations of these five lengths for five phases, but only 70 of them lead to a series length of 19 (i.e., there are 70 randomizations in this case).

## An example

**Fig. 3:**
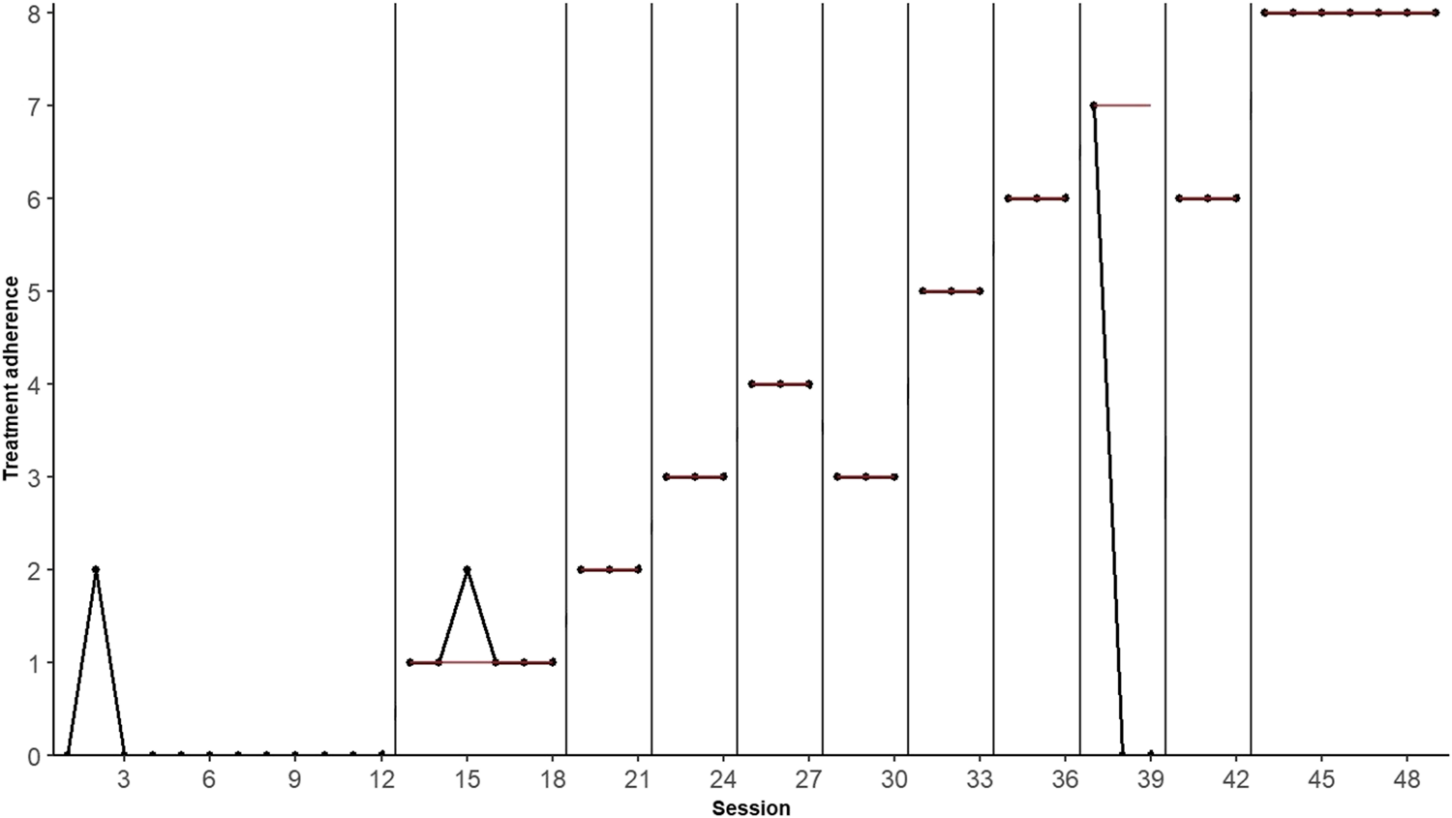
Data from patient Ann in a criterion designed used by Fitterling et al. (1988) to assess the effectiveness of behavioral management of exercise training in vascular headache patients

The following example is taken from a study conducted by Fitterling et al. (1988), who used a CCD to shape and maintain treatment adherence to aerobic exercise training in five patients with vascular headache. Each subject was provided with a personalized exercise prescription for three sessions per week. The primary dependent variable was self-reported aerobic exercise behavior, which was assessed using Cooper points, a “standardized measure of the amount of aerobic benefit derived from different exercise topographies, intensities, and durations” (Fitterling et al., 1988, p. 11). The earned Cooper points, ranging from 0 to 8, were then compared to the criteria set by the researchers throughout the study, which included reversals to lower criteria to demonstrate experimental control. Figure 3 shows the data for participant Ann, a 38-year-old female with vascular headache onset at age 13.

Ann’s data contain 49 measurements, consisting of a baseline phase with 12 measurements and 37 experimental measurements distributed over 10 criteria. The following paragraphs explain how PCM and BAC use this information differently to construct the randomization distribution.

### Phase change moment randomization (without baseline)

If the $n=37$ measurements and each of the $I=10$ phases—excluding the baseline as previously explained—should have at least $k=3$ data points, and there are $I-1=9$ phase change moments, then there are $$\left(\begin{array}{c}n-I*k+(I-1)\\ (I-1)\end{array}\right)=\left(\begin{array}{c}37-10*3+9\\ 9\end{array}\right)=$$ 11,440 possible randomizations, following Onghena’s (1992) formula. Below is a non-exhaustive list of combinations of possible phase change moments: each number refers to the measurement occasions considering that the first 12 measurements belong to the baseline and the first intervention phase starts on the 13th measurement occasion. Thus, the first number indicates the phase change from first to second criterion. The combination actually used in the experiment is marked in bold:

- **19, 22, 25, 28, 31, 34, 37, 40, 43**
- 16, 19, 22, 25, 28, 31, 34, 37, 40
- 16, 19, 22, 25, 28, 31, 34, 37, 41
- 16, 19, 22, 25, 28, 31, 34, 37, 42
- …
- 21, 26, 29, 32, 35, 38, 41, 44, 47
- 22, 26, 29, 32, 35, 38, 41, 44, 47
- 23, 26, 29, 32, 35, 38, 41, 44, 47

**Fig. 4:**
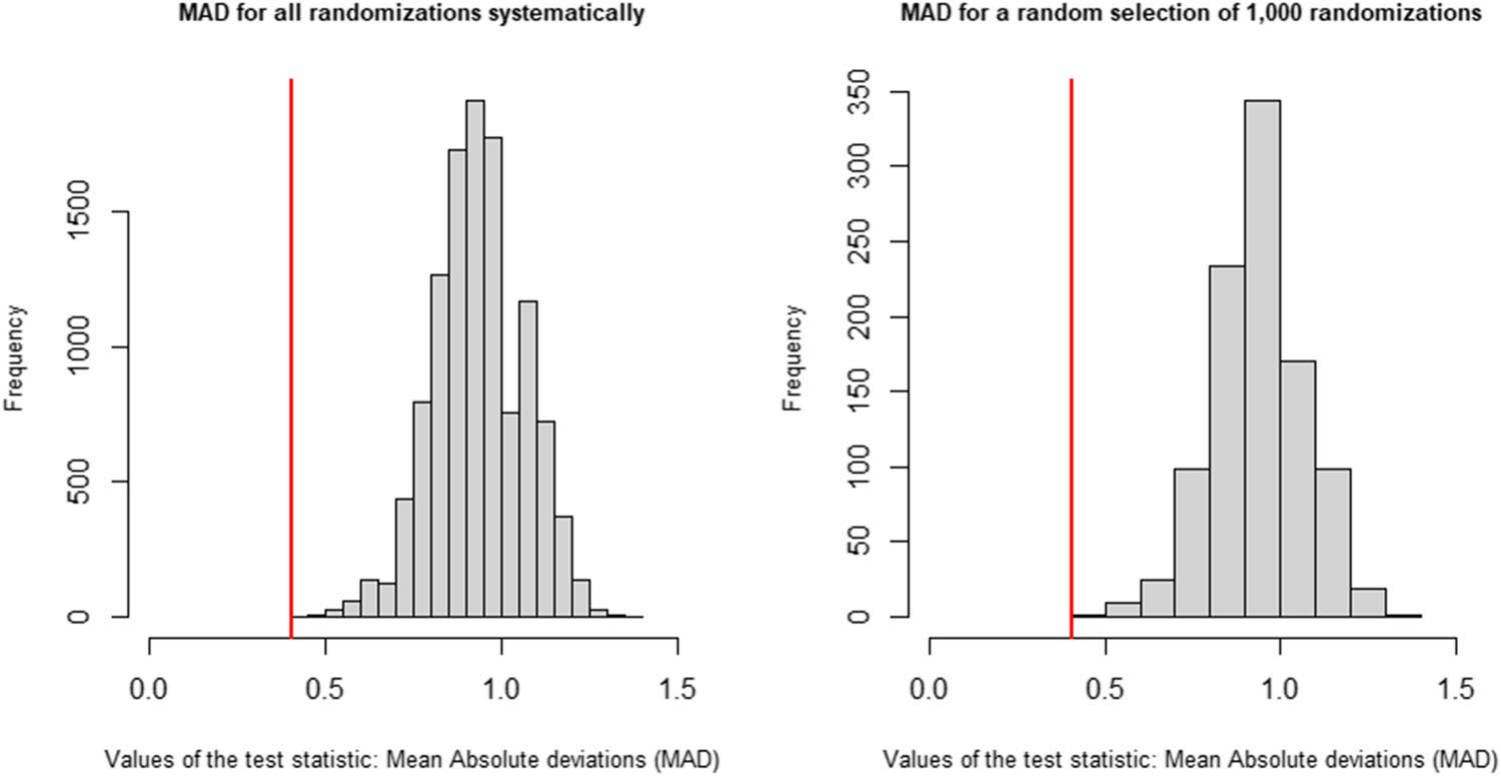
Randomization distribution under the phase change moment (PCM) randomization procedure, using a Monte Carlo sample of all possible randomizations (left panel) and all possible randomizations (right panel)*. Note.* The observed test statistic is indicated by the red vertical line

It is possible to list all randomizations systematically. Alternatively, to reduce computational time, which can become quite long for a large number of randomizations even with present-day computers, a Monte Carlo random sample can be taken. Generally, a Monte Carlo random sample of 1000 randomizations suffices to reach adequate statistical power (Edgington, 1969). The value of the observed test statistic for the actual randomization (i.e., actual phase lengths) is 0.41, indicated by the red vertical line in Fig. 4. According to the systematic randomization (Fig. 4, left panel), the *p*-value is <.001, as the observed MAD is the smallest of all 11,440 MAD values. Similarly, according to Monte Carlo randomization (Fig. 4, right panel), the *p*-value is .001, as the observed MAD is also the smallest of all 1,000 MAD values obtained for the random Monte Carlo samples of all possible randomizations. With the PCM procedure, the null hypothesis of no treatment effect can thus be rejected at the conventional level of $\alpha =.05$.

### Blocked alternating criterion randomization

With the BAC procedure, the 10 experimental criteria can be segmented into five blocks of two criteria each. Within each block—indicated by square brackets—the order of criterion implementation can then be determined randomly. There are ${2}^{5}=32$ possible orders of criterion implementation, with the one actually used in the experiment marked in bold:

- **[1,2], [3,4], [3,5], [6,7], [6,8]**
- [2,1], [3,4], [3,5], [6,7], [6,8]
- [2,1], [4,3], [3,5], [6,7], [6,8]
- …
- [2,1], [4,3], [5,3], [6,7], [6,8]
- [2,1], [4,3], [5,3], [7,6], [6,8]
- [2,1], [4,3], [5,3], [7,6], [8,6]

**Fig. 5:**
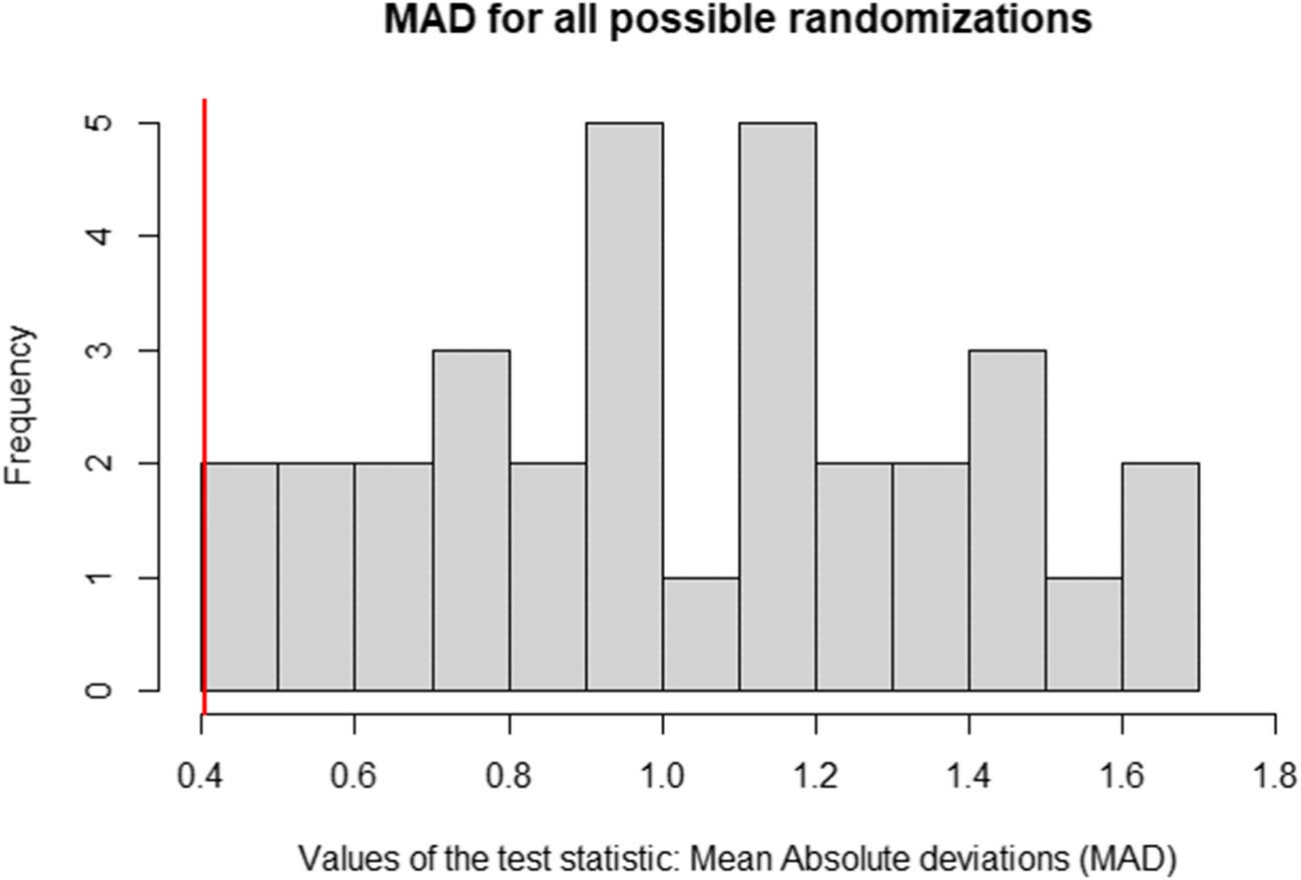
Randomization distribution under the blocked alternation randomization procedure. *Note.* The observed test statistic is indicated by the red vertical line

The value of the observed test statistic (i.e., for the actual order of criterion levels used in the experiment) is 0.41. This is the smallest MAD for the 32 possible randomizations, and thus, $p = 1/32=.03$. The randomization distribution is presented in Fig. 5. The observed test statistic is indicated by the vertical red line. With the BAC procedure, the null hypothesis of no treatment effect can also be rejected at the conventional level of $\alpha =.05$.

## Method

### Literature review for informing the simulation conditions

In order to determine the simulation conditions, we performed a literature review, using the references from Manolov et al. (2020), who extended the review of CCDs by Klein et al. (2017). A total of 129 articles were consulted (as online supplementary material we provide Appendix A available at https://osf.io/2utqm/ containing the references to these articles). In case an article included more than one CCD plot with data, we only used the first plot, in order to have all studies equally represented (except for Schleien et al., 1981, in whose study there was only one criterion level for the first participant). If a criterion level was discontinued to return to baseline and afterwards reinstated (e.g., Kowalewicz & Coffee, 2014; McDaniel & Bruhn, 2016), this was counted as two separate intervention subphases (not counting the intermediate baseline phase). If a design included several intervention phases, and each of these phases included several criteria (e.g., Luiselli et al., 2013), each of these criteria was considered as a separate intervention phase.

In relation to the number of measurements, we coded the following aspects: (a) presence of an initial baseline phase and number of baseline measurement occasions; (b) number of criterion phases; and (c) number of measurements for each of the intervention phases. As a consequence of the latter, we quantified the proportion of studies in which all intervention phases were of the same length. For 14 of the 129 studies, after reading the text and inspecting the plots (where available), we were not able to exactly tally the number of measurements for each intervention phase, although for some of these studies it was clear from the plots that certain intervention phases were longer than others.

According to the results of this coding (Table 1), 15% of the CCD data sets reviewed had all intervention phase lengths of the same size. The typical intervention phase length was found to be between three and six measurements (percentiles 25 and 75, respectively), while guidelines actually recommend at least five data points per intervention phase (What Works Clearinghouse, 2022). The number of criterion phases was found to be typically between four and seven (percentiles 25 and 75, respectively), which aligns better with the What Works Clearinghouse guideline requiring at least three criterion changes (i.e., four criteria).

**Table 1:** Results of the review of published empirical studies using changing criterion designs

	Baseline length	Criterion phases	Criterion phase lengths
Minimum	0.00	2.00	1.00
Percentile 10	1.00	3.00	2.00
Percentile 25	3.00	4.00	3.00
Median	4.00	5.00	4.00
Mean	5.23	6.14	5.81
Percentile 75	6.25	7.00	6.00
Percentile 90	11.00	12.00	11.00
Maximum	36.00	20.00	87.00

In relation to the criterion levels, we coded the type of criterion used in relation to the target behavior and also the specific criterion levels employed. In relation to the former, the following categories were used: percentage completion or percentage of intervals in which the target behavior takes place; frequency or rate (e.g., per minute) of the target behavior (including number of steps performed in a given task); duration or latency (e.g., in seconds or minutes); and other (which included a variety of measures such as test scores, ratings, amount of substance, distance in feet, weight in pounds, loudness in decibels, and muscle tension in microvolts). In 46% of the studies, a frequency or a rate was recorded, with the most common situation being increases of one step across criterion levels. In approximately 21% of the studies, a percentage of completion or percentage of intervals was the measure used, with typical difference between criterion levels being around 10–25%. In approximately 17% of the studies, duration or latency was the focal measure, with a typical difference between criterion levels being of 5–10 units (in seconds or minutes).

### Simulation conditions for PCM

Given that PCM and BAC lead to a different number of possible randomizations for the same series lengths and number of phases, it was decided to have somewhat different simulation conditions for the two randomization procedures, in order to ensure that (a) there are enough randomizations for rejecting a null hypothesis at the .05 level, and (b) the randomization test is computationally feasible in terms of computation time and coding.

For PCM, the number of intervention phases ranged between three (percentile 10 in our review, as per Table 1) and seven (percentile 75). The minimum phase length (which needs to be pre-established when randomizing under PCM) was set to three. This minimum is related both to (1) a bare minimum requirement for minimal phase lengths that has been mentioned in methodological quality guides (Tate et al., 2013; What Works Clearinghouse, 2022); (2) the common use of mastery criteria such as three consecutive sessions at the criterion level (Manolov et al., 2020); and (3) the results of the conducted review. The intervention phase lengths ranged from three (percentile 25) to seven (beyond percentile 75). The total series lengths ranged from 15 to 42. The combination of seven phases and a phase length of seven (i.e., a series length of 49) was not studied, due to computational issues in obtaining either the systematic listing of all 906,129 possible randomizations or a Monte Carlo sample of combinations of seven phase lengths (with a minimum of three and a maximum of 29) leading to a series length of 49.

When performing a simulation on PCM, in addition to the computational challenges mentioned in the “Similarities and Key Differences between the Procedures” section, another complication arises. Specifically, a complication that is not present when analyzing an actual data set. When there is an actual series of, for example, 20 measurements, belonging to four phases with lengths 3, 6, 5, 6, the MAD value for this combination of phase lengths will be compared to all 165 MAD values that can be obtained under PCM, in order to obtain the *p*-value. However, when a simulation is performed, there is not a single combination of phase lengths that can be considered the “actual data.” In fact, it is possible to obtain estimates of type I error rates and statistical power for all 165 possible randomizations, as if they were the actual data set. Such studies have been carried out for randomization tests under the label “data-division-specific” type I error and power for reversal designs (Manolov & Solanas, 2008; Manolov et al., 2010) and for AB designs (Solanas et al., 2008). Given that it is not feasible to study or report such specific estimates for each possible combination of phase lengths, for each possible total series length and number of phases, we had to choose which randomization to treat as the “actual data” when performing the simulations. On the basis of the intervention phase lengths observed in our literature review (see the Excel file at https://osf.io/pq5kf/), we decided to focus on three patterns of phase lengths: uniform (i.e., all phases are of the same size), increasing (i.e., later phases are longer), and triangular (i.e., middle phases are longer than earlier and later phases).

### Simulation conditions for BAC

For BAC, the number of criterion levels per block was set to 2. To be able to obtain a *p*-value as small as .05, at least five blocks are required (i.e., ten intervention phases). We simulated conditions with the number of phases ranging from 10 to 20 (with the latter being the maximum observed in our literature review), not taking into account the baseline. All phases were simulated to be of the same size because the phase length does not influence the number of randomizations for BAC, with phase lengths ranging from three to six, as for PCM. Thus, the shortest series length simulated was 30 (ten phases, each with three measurements) and the longest was 120 (20 phases, each with six measurements).

### Simulation conditions common to both PCM and BAC

#### Intervention effect conditions

For studying statistical power, criterion changes were introduced across intervention phases. Specifically, the initial plan was to implement a one-unit increase (representing frequencies or steps when performing a target behavior), a five-unit increase (representing duration, typically in seconds), or a ten-unit increase (representing percentages of completion), with all values being derived from the previously described review of CCDs. The baseline level was equal to 10.

Random variability was simulated around the criterion levels established for each phase. This variability was programmed to represent 10%, 25%, and 50% of the specific criterion level. In that sense, larger criterion levels were associated with greater variability, and thus this variability was not constant for the whole data series.

The initial planning was then modified. Once we started obtaining results of the simulation, it was discovered that the amount of increase in the criterion levels (and the corresponding increase in the data generated for different phases) did not affect statistical power for both procedures, given that the random variability was simulated in relation to the criterion levels. In that sense, under conditions with an increase of five units, a phase with a criterion of 15 is followed by a phase with a criterion of 20, and the amount of random variability is (in conditions with 10% random error) is 1.5 and 2, respectively. Under conditions with an increase of one unit, a phase with a criterion of 15 is followed by a phase with a criterion of 16, and the amount of random variability is (in conditions with 10% random error) 1.5 and 1.6, respectively. Thus, the proportionate increase in random variability associated with a greater difference between criterion levels led to the power estimates being practically identical for an increase of one or five units. For example, for the BAC procedure and data generated with ten phases, three measurements per phase, positive autocorrelation of .3, and random variability of 10%, the power for an increase of 1 was .74, and for an increase of 5 it was .72. Therefore, in the Results section, we focus on an increase of five units.

#### No-effect condition

In order to study type I error rates, the data were simulated at a constant level. That is, there were no shifts programmed in the data between any of the phases. In order to represent the fact that they belong to different criterion phases, the same criterion levels were used as for the intervention effect conditions. Thus, when the null hypothesis is true, there is a mismatch between the data (which include no change) and the criterion levels (which are different across phases). This is equivalent to simulation studies for studying type I error rates for SCEDs entailing A–B comparisons: the data incorporate no difference, but are treated as belonging to different phases. Specifically, the mean level of the data was simulated to be equal to the average of all criterion levels. For instance, if the criterion levels used for computing MAD are 5, 10, 15, and 25 for four phases, respectively, the mean level of the data is simulated to be the average of 5, 10, 15, and 25 (that is, 12.5). For PCM, a weighted average of the criterion levels is then used, with the weight being the number of measurements in each phase. For BAC, all phases had the same number of measurements, and thus the weight of each criterion level was equal when determining the average level of the simulated data series.

Random variability was simulated around this average level. Specifically, this random variability was defined as a proportion of the average level: 10%, 25%, and 50%. Given that, to the best of our knowledge, there are no previous stimulation studies on CCDs, it was not possible to replicate values from existing research. Instead, we chose values that would represent lower and higher degrees of variability. Moreover, when carrying out the simulations, it was verified that 10% random variability was associated with a ceiling effect (i.e., power estimates close or equal to 1) in most simulation conditions, whereas 50% random variability was associated with values close to the nominal alpha in some simulation conditions (e.g., for the BAC procedure and data generated with ten phases, three measurements per phase, and positive autocorrelation of .3, the power for an increase of 5 was .09). The amount of random variability was constant throughout the whole data series, as the average level was also constant in the no-effect conditions.

#### Common to conditions with and without an effect

Regarding autocorrelation, both independent data (zero autocorrelation), positive serial dependence (0.3) and negative (−0.3), were simulated. This aligns well with the review of SCED research by Shadish & Sullivan (2011), who reported an average estimate of approximately −0.1 for CCDs, but also considering that an estimate of the average does not entail that all autocorrelations are close to zero (see, for instance, the review by Solomon, 2014).

Mini-reversals were programmed in some conditions, following the recommendations by Klein et al. (2017). Specifically, for conditions with at least four intervention phases, the penultimate phase entailed a reversal to a previous criterion (e.g., a decrease of five units), whereas the last phase again entailed an increase, just as the initial phases entailed increases in the criterion level. For BAC, these reversals were programmed only for a phase length of three measurement occasions. For PCM, the reversals were also programmed only for some conditions: specifically, for one condition per number of phases (a series length of 16 when there are four phases, a series length of 20 when there are five phases, a series length of 24 when there are six phases, and a series length of 28 when there are seven phases). A summary of the simulation conditions is presented in Table 2.

**Table 2:** Summary of the simulation conditions

Blocked alternating criterion	
Blocks of two phases	5, 6, 7, 8, 9, 10
Phases	10, 12, 14, 16, 18, 20
Phase lengths	3, 4, 5, 6
Series lengths	30, 36, 40, 42, 48, 50, 54, 56, 60, 64, 72, 80, 84, 90, 96, 100, 108, 120
Phase change moment	
Phases	3, 4, 5, 6, 7
Phase lengths	3, 4, 5, 6, 7 (for uniform phase length pattern)
Series lengths	15, 16, 18, 20, 21, 24, 25, 28, 30, 35, 36, 42
Common to both blocked alternating conditions and phase change moment	
Autocorrelation	−.3, 0, .3
Random variability	10%, 25%, 50%
Change in the data across criterion phases	0, 5

A total of 1000 iterations per simulation condition were used, as in Michiels & Onghena (2019); Michiels et al. (2020). This is more than the 500 iterations used in Bouwmeester & Jongerling (2020) and less than the 10,000 iterations used by Levin et al. (2017, 2018, 2021). We performed 10,000 iterations for some conditions, and the similarity in the estimates obtained to those based on 1000 led us to decide to prioritize computational efficiency. The R code for the simulations is available at the Open Science Framework project: https://osf.io/dbfxk/.

### Data analysis

MAD was used as test statistic, computing it for the data under the actual phase lengths (PCM procedure) or criterion levels (BAC procedure) and also under all possible randomizations according to the randomization procedure. The *p*-value was calculated as the number of test statistics as small or smaller than the actual one, divided by the total number of test statistics in the randomization distribution.

Two main aspects were the object of analysis. On the one hand, the type I error rate refers to the proportion of iterations in which the randomization test *p*-value is equal to or less than .05 in the no-effect condition. On the other hand, statistical power refers to the proportion of iterations in which the randomization test *p*-value is equal to or less than .05 in the intervention effect conditions. The estimates of both type I error rates and statistical power were based on systematic listing of all possible randomizations for BAC. For PCM, we used either systematic randomizations (up to series length of 36 measurements) or 1000 random Monte Carlo samples from all possible randomizations for each simulation condition for series with 42 measurements.

Once the type I error rates and statistical power estimates were available, analyses of variance (ANOVAs) were performed for identifying the factors that contribute the most to the variation in type I error rates and power. Specifically, the factors were series length, number of phases, number of randomizations, random variability, autocorrelation, and type of data pattern (for PCM). We studied the main effect of each of these factors via one-way ANOVAs, and we also studied second-order interaction effects from two-way ANOVAs crossing the factors number of randomizations, series length, random variability, autocorrelation, number of phases (for PCM) or number of blocks (for BAC), and phase length pattern and presence/absence of reversal for PCM. The focus was placed on the eta-squared values, using as benchmarks .01–.05 (small effect), .06–.14 (medium effect), and .15–1.00 (large effect), rather than on the statistical significance of the ANOVAs. This is consistent with the analyses performed in previous simulation studies in which several simulation parameters were manipulated (Baek & Ferron, 2020; Declercq et al., 2021; Jamshidi et al., 2021; Joo et al., 2019).

For studying the effect of including a reversal phase in the design, we applied paired-samples *t*-tests, comparing the same simulation conditions (series length, number of phases, degree of autocorrelation, amount of random variability) with and without reversal. From these *t*-tests, we computed an *R*-squared value using the formula from Fritz et al. (2012): ${r}^{2}={t}^{2}/({t}^{2}+\nu )$, where $\nu$ is the degrees of freedom. This *R*-squared value is interpreted the same as the eta-squared, as a proportion of variability (in the type I error or power estimates) as a function of the simulation factor (here, the presence or absence of a reversal).

## Results

The full results of the simulation can be downloaded from https://osf.io/dbfxk/, whereas in the current section we will review the main findings. The effect of the simulation conditions on the estimates of type I error rates and statistical power is summarized in Tables 3 (main effects) and 4 (second-order interactions), which expresses the strength of association between each simulation factor and these estimates as a proportion of variability explained (i.e., an eta-squared or an *R*-squared value).

**Table 3:** Summary of the main effect of the simulation conditions on the estimates of type I error rates and statistical power, expressed as eta-squared values or R-squared values (for reversal only)

Factors for BAC	Type I error	Statistical power
*R*	0.10	0.03
*B*	0.10	0.03
*n_p*	0.05	0.02
*n*	0.18	0.05
*Var*	0.35	0.92
*φ*	0.00	0.02
Reversal (yes or no)	0.01	0.14
Factors for PCM	Type I error	Statistical power
*R*	0.26	0.08
*I*	0.07	0.01
*n*	0.11	0.06
*Var*	0.00	0.86
*φ*	0.00	0.01
Reversal (yes or no)	0.13	0.51
Phase length pattern	0.13	0.00

*Note. BAC –* blocked alternating conditions. *PCM –* phase change moment.* R* – number of randomizations.* n* – total number of measurements (series length). *n*_*p*_ – number of measurements per phase. *φ* – degree of autocorrelation. *Var* – random variability. *B* – number of blocks when using blocked alternating conditions randomization. *I* – number of phases when using phase change moment randomization

In light of the presence of autocorrelation in SCED data (Barnard-Brak et al., 2021; Shadish & Sullivan, 2011; Sideridis & Greenwood, 1997; Solomon, 2014) and its prominence in SCED simulation studies on different data analytical procedures (e.g., Baek & Ferron, 2013; Bishara et al., 2021; De & Onghena, 2022; Hedges et al., 2023; Petit-Bois et al., 2016; Smith et al., 2012), including randomization tests (Bouwmeester & Jongerling, 2020; Ferron & Onghena, 1996; Ferron & Sentovich, 2002; Levin et al., 2012; Manolov, 2019), it was decided to segment the results per level of autocorrelation in Figs. 8, 9, 10, 11, 12, and 13.

### Type I error rates

For both PCM and BAC, type I error rates are very low. For most simulation conditions, the estimates are below .05. The only exception is for PCM and a triangular pattern of phase lengths for series lengths of 20 (and five phases), 24 (and six phases), and 28 (and seven phases).

**Fig. 6:**
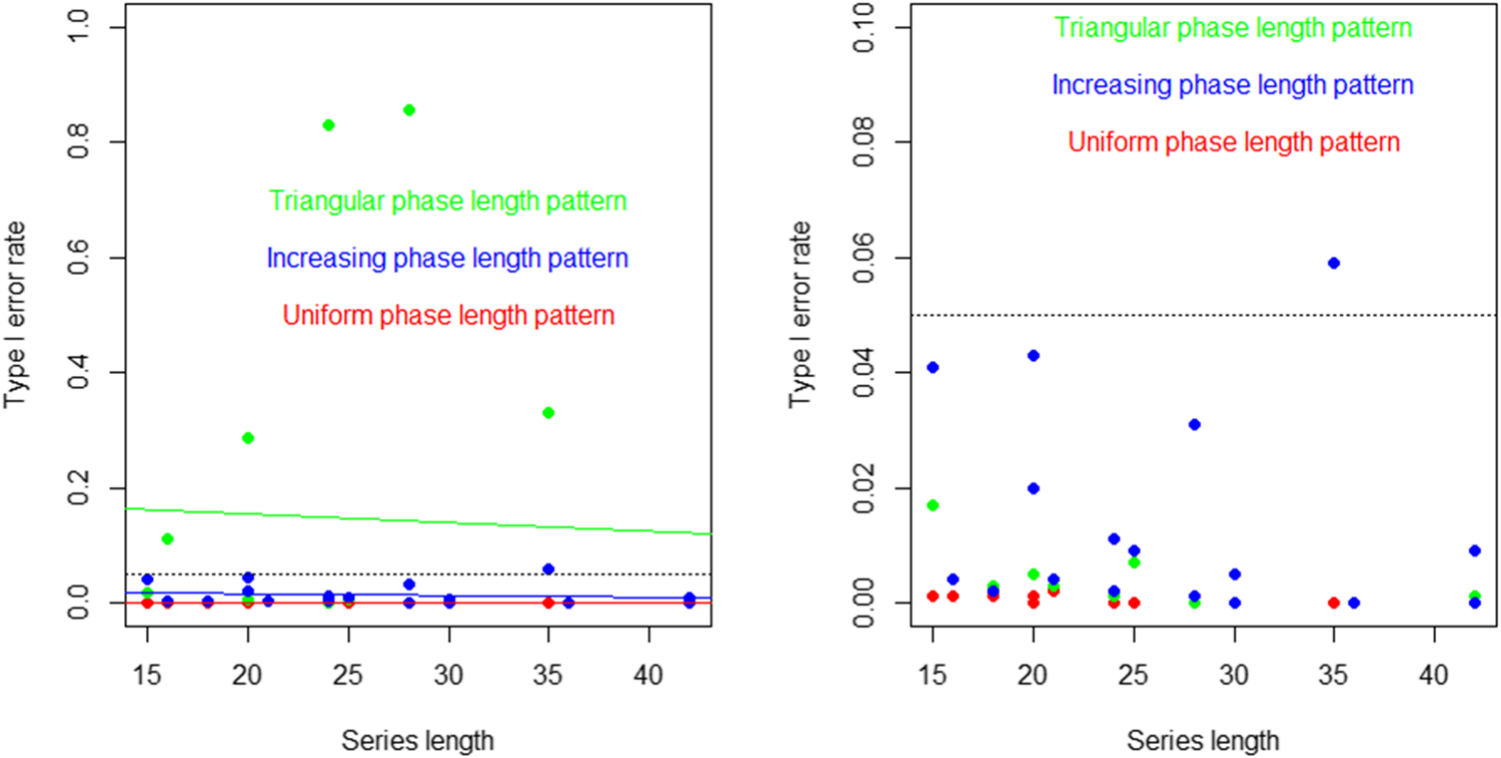
Relation between series length and the phase length pattern (green: triangular; red: uniform; blue: increasing) and type I error rates for the phase change moment (PCM) randomization procedure. The results refer to data with no serial dependence and a random variability of 25%. The right panel is a zoom of the left panel, focusing on type I error rates between 0 and .10

Regarding the factors affecting the type I error rates, for PCM, the number of randomizations and the phase length pattern has the strongest effect (eta-squared equal to .26 and .13, respectively, as per Table 3). Figure 6 provides an illustration of the relation between series length (or lack thereof), the type of pattern of combinations between phase lengths, and type I error rates. As per Table 4, the interaction effect is strongest between the phase length pattern and the number of randomizations measurements (${\eta }^{2}$=.49), the phase length pattern and the number of measurements (${\eta }^{2}$=.19), and the phase length pattern and the number of phases (${\eta }^{2}$=.12), in relation to the worse results for the triangular pattern when there are more phases (between 5 and 7) and an intermediate total number of measurements (between 20 and 28), leading to a certain number of randomizations (126 and 1716). The left panel includes the whole span of estimates, illustrating the excessively high values for the triangular pattern, whereas the right panel zooms in the area with estimates between 0 and .10. The commonly used .05 threshold is depicted with a dotted horizontal line.

**Table 4:** Summary of the interaction effect of the simulation conditions on the estimates of type I error rates and statistical power, expressed as eta-squared values

Factors for BAC	Type I error	Statistical power
*n* x *B*	0.00	0.00
*φ* x *B*	0.02	0.00
*φ* x *n*	0.08	0.00
*n* x *Var*	0.16	0.01
*B* x *Var*	0.07	0.00
*φ* x *Var*	0.01	0.00
*R* x *φ*	0.02	0.00
*R* x *Var*	0.07	0.00
reversal x *R*	0.00	0.00
reversal x *n*	0.00	0.00
reversal x *B*	0.00	0.00
reversal x *φ*	0.00	0.00
reversal x *Var*	0.00	0.00
Factors for PCM	Type I error	Statistical power
*n* x *I*	0.01	0.00
*φ* x *I*	0.00	0.00
*φ* x *n*	0.00	0.00
*n* x *Var*	0.01	0.02
*I* x *Var*	0.00	0.00
*φ* x *Var*	0.00	0.00
*R* x *φ*	0.00	0.00
*R* x *Var*	0.03	0.03
*R* x pattern	0.49	0.00
*n* x pattern	0.19	0.00
*I* x pattern	0.12	0.00
*φ* x pattern	0.00	0.00
*Var* x pattern	0.02	0.00
reversal x *R*	0.06	0.01
reversal x *n*	0.05	0.01
reversal x *I*	0.06	0.01
reversal x *φ*	0.00	0.00
reversal x *Var*	0.01	0.01

*Note. BAC –* blocked alternating conditions. *PCM –* phase change moment. *R* – number of randomizations.* n* – total number of measurements (series length). *φ* – degree of autocorrelation. *Var* – random variability. *B* – number of blocks when using blocked alternating conditions randomization. *I* – number of phases when using phase change moment randomization

**Fig. 7:**
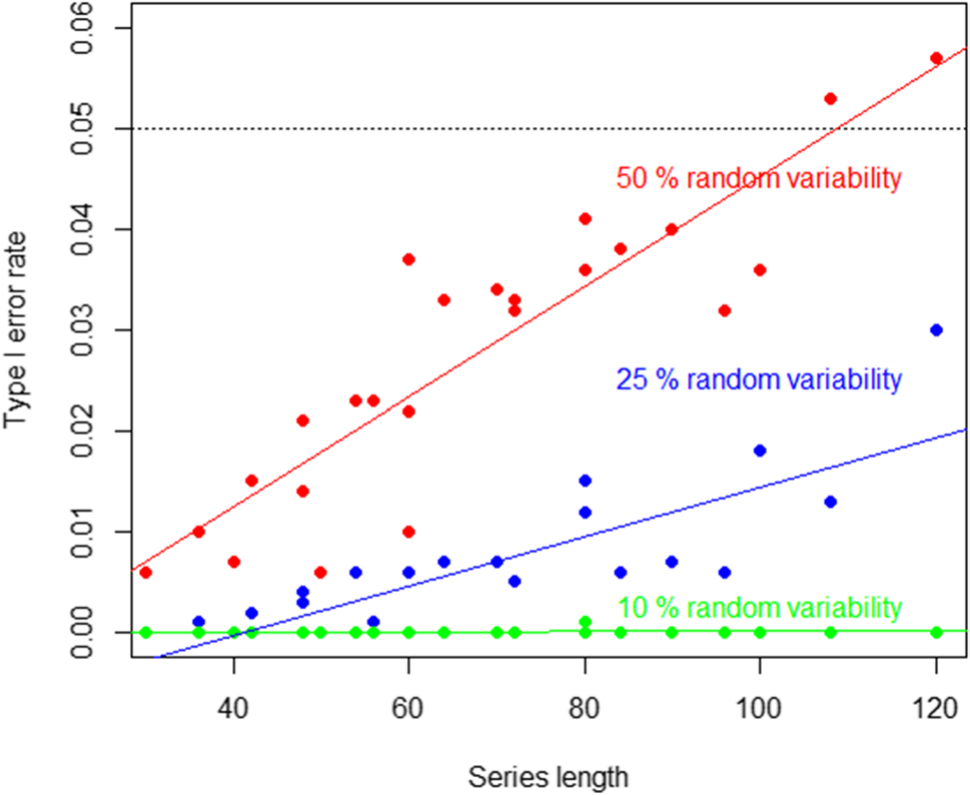
Relation between series length and the amount of random variability (green: 10%; red: blue: 25%; red: 50%) and type I error rates for the blocked alternating conditions (BAC) randomization procedure. The results refer to data with no serial dependence

For BAC, the factors most strongly related to the type I error rates were the amount of random variability and series length (eta-squared equal to .35 and .18, respectively, as per Table 3). Figure 7 provides an illustration. Note that having longer series is related also to having more phases and blocks, although the effect of the latter two on type I error rates was not as strong as the effect of series length, as shown in Table 3. The interaction between the series length and the amount of random variability yielded an eta-squared value of .16, whereas for the interaction between the number of blocks and the amount of random variability, ${\eta }^{2}=.07$ (see Table 4), illustrating the fact that greater random variability is associated with higher type I error rates when there are more blocks and measurements. The presence of positive or negative autocorrelation did not have any relevant effect on type I error rates for either PCM or BAC.

Regarding the effect of the presence of a reversal, it is only noteworthy for PCM, with an eta-squared value of .13. Specifically, in the presence of reversals, the problematic type I error rates observed for triangular phase length patterns are practically not present (i.e., the type I error rates are controlled).

### Statistical power

#### Factors affecting power

For both PCM and BAC, power was close to 1 when the random variability was 10% of the criterion level in place for each phase. When the random variability was 25%, power was in general larger than .50; for PCM and series lengths of 28 (and four phases), 30 (and five phases), 36 (and six phases), and 42 (and six or seven phases). In many simulation conditions, power reached .80 even for a random variability of 25%. In contrast, for random variability of 50%, all power estimates are below .50, and most are below .30.

Regarding the factors affecting statistical power, for both PCM and BAC, the amount of random variability has the strongest effect (eta-squared equal to .87 and .92, respectively). For PCM, the second strongest predictor is the number of combinations of phase lengths (${\eta }^{2}=.08$). For BAC, the second strongest predictor is series length (${\eta }^{2}=.05$). Note again that having longer series is related also to having more phases and blocks, although the effect of the latter two on the power estimates was not as strong as the effect of series length, as shown in Table 3. None of the second-order interactions of factors account for any substantial variability in statistical power (i.e., all ${\eta }^{2}\le .02$ in Table 4), for either PCM or BAC.

Regarding the effect of the presence of a reversal, it is especially salient for PCM (${\eta }^{2}=.50$), but it is also present for BAC (${\eta }^{2}=.14$). Specifically, in the presence of reversal, power is higher (.09 on average for PCM and .01 on average for BAC).

#### Statistical power for PCM: Further detail

**Fig. 8:**
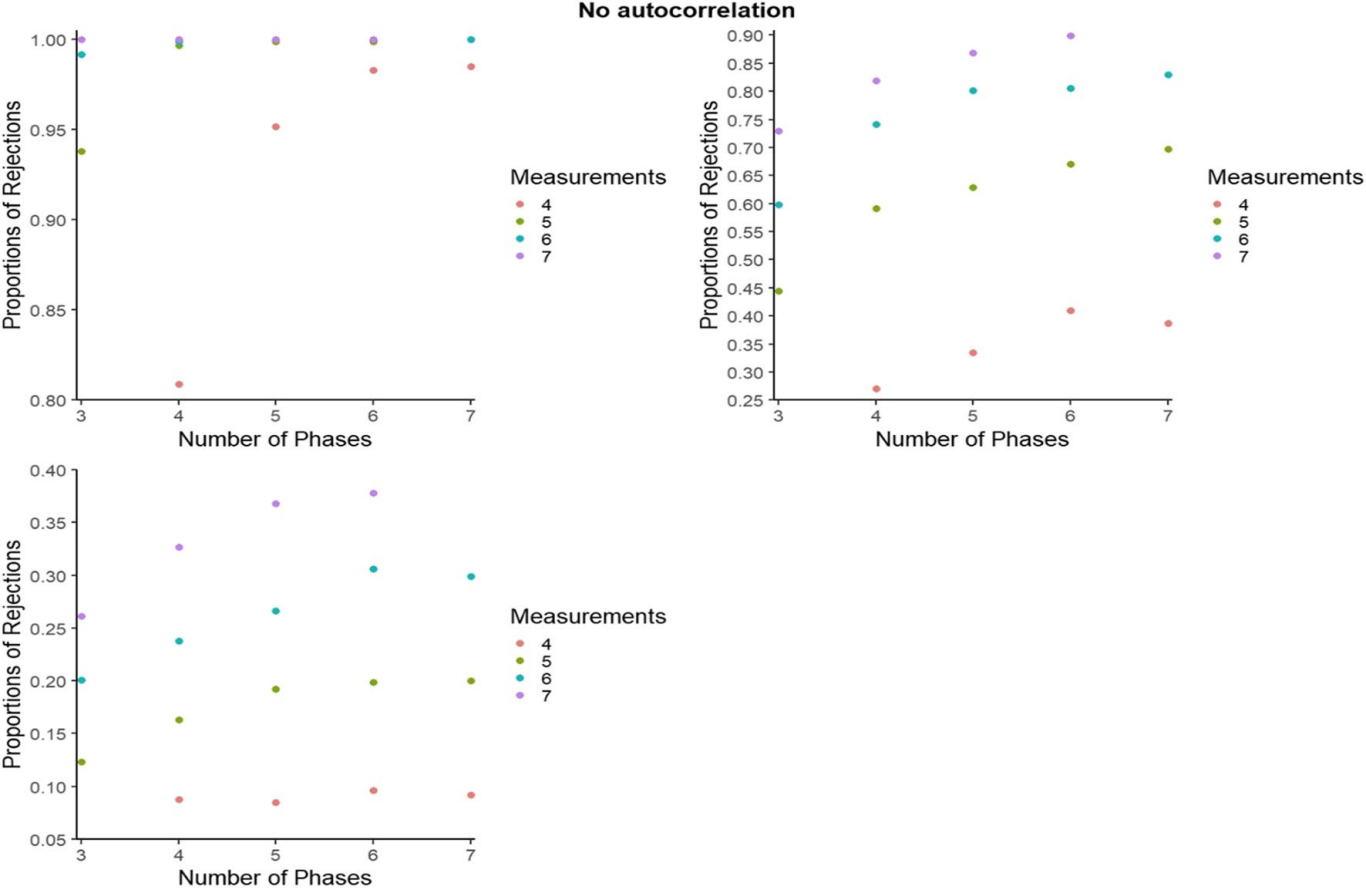
Statistical power estimates for phase change moment randomization, according to the number of phases and the number of measurements per phase. Independent data. Random variability: upper left - 10%, upper right - 25%, bottom - 50%

**Fig. 9:**
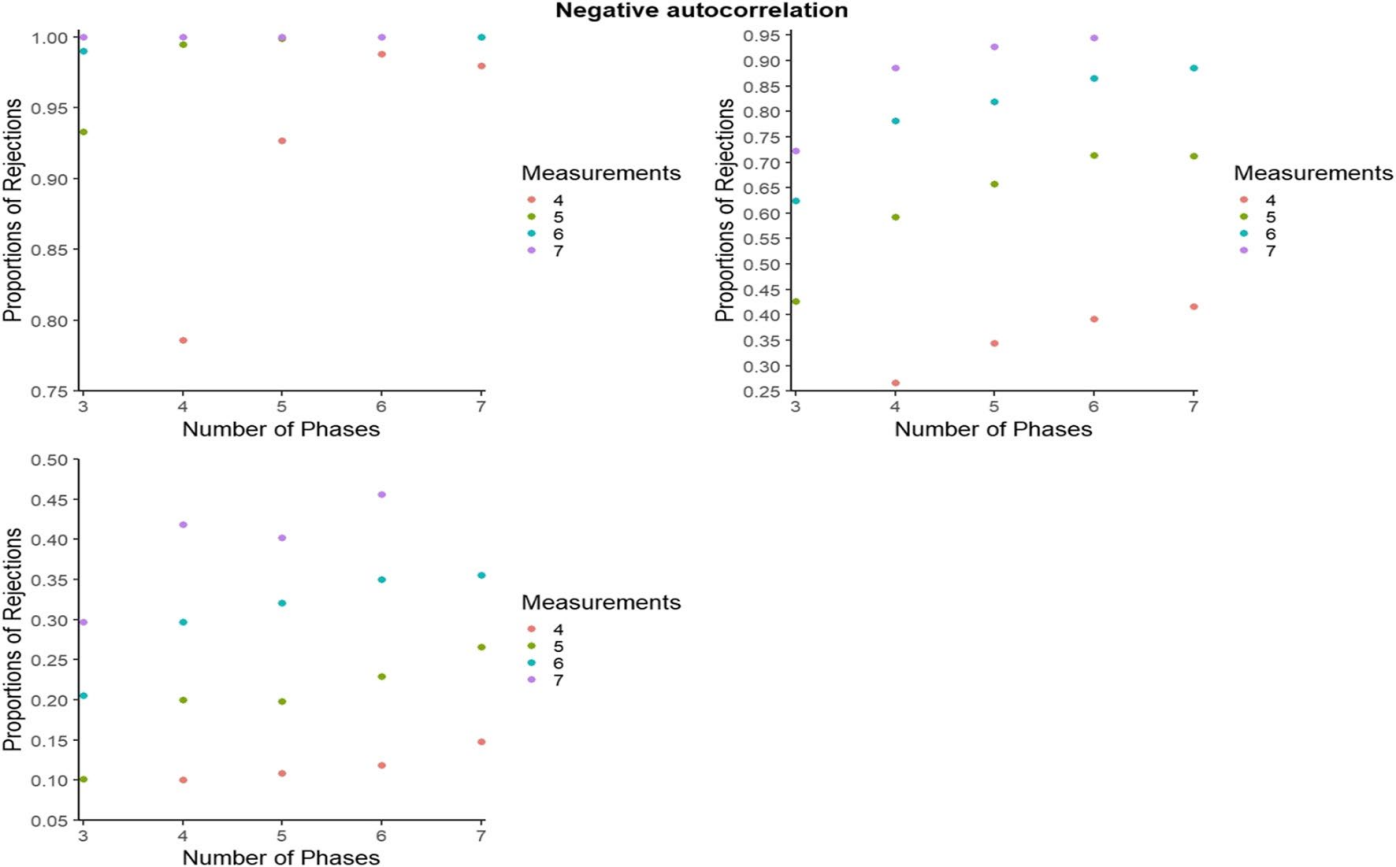
Statistical power estimates for phase change moment randomization, according to the number of phases and the number of measurements per phase. Data with negative autocorrelation. Random variability: upper left - 10%, upper right - 25%, bottom - 50%

**Fig. 10:**
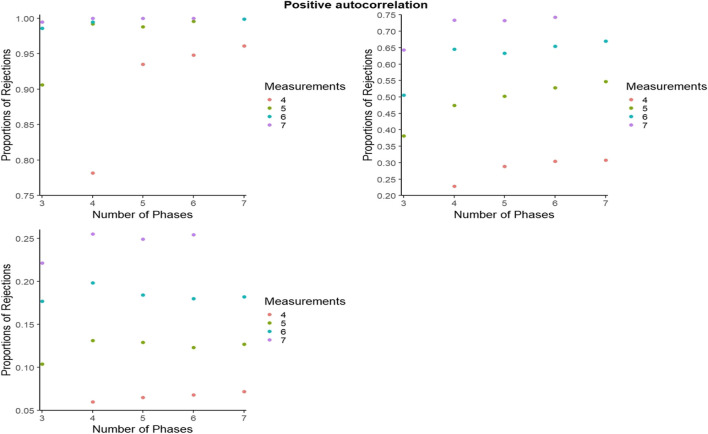
Statistical power estimates for phase change moment randomization, according to the number of blocks and the number of measurements per phase. Data with positive autocorrelation. Random variability: upper left - 10%, upper right - 25%, bottom - 50%

For representing the statistical power for PCM, to avoid excessive cluttering and to better zoom in on the differences within and between the number of phases and number of measurements per phase, the *y*-axes were set separately for each panel. However, figures with axes ranging from 0 to 1 can be consulted from the online supplementary material called Appendix B, available from https://osf.io/hry2f. Figures 8, 9, and 10 refer to independent data, negative autocorrelation, and positive autocorrelation, respectively.

In general, statistical power is lowest for positive autocorrelation for all simulation conditions, whereas power increases with the number of measurements per phase. The largest increase in power appears when increasing the number of measurements per phase from 4 to 5. In the presence of low variability (10%), power reaches 1 when there are at least six measurements per phase, regardless of the number of phases. In the presence of low variability (10%) and for independent data, power is at least .80 as long as there are at least four phases. When there is more variability (25%), power reaches .80 when there are seven measurements per phase and the data are independent or present a negative autocorrelation.

#### Statistical power for BAC: Further detail

**Fig. 11:**
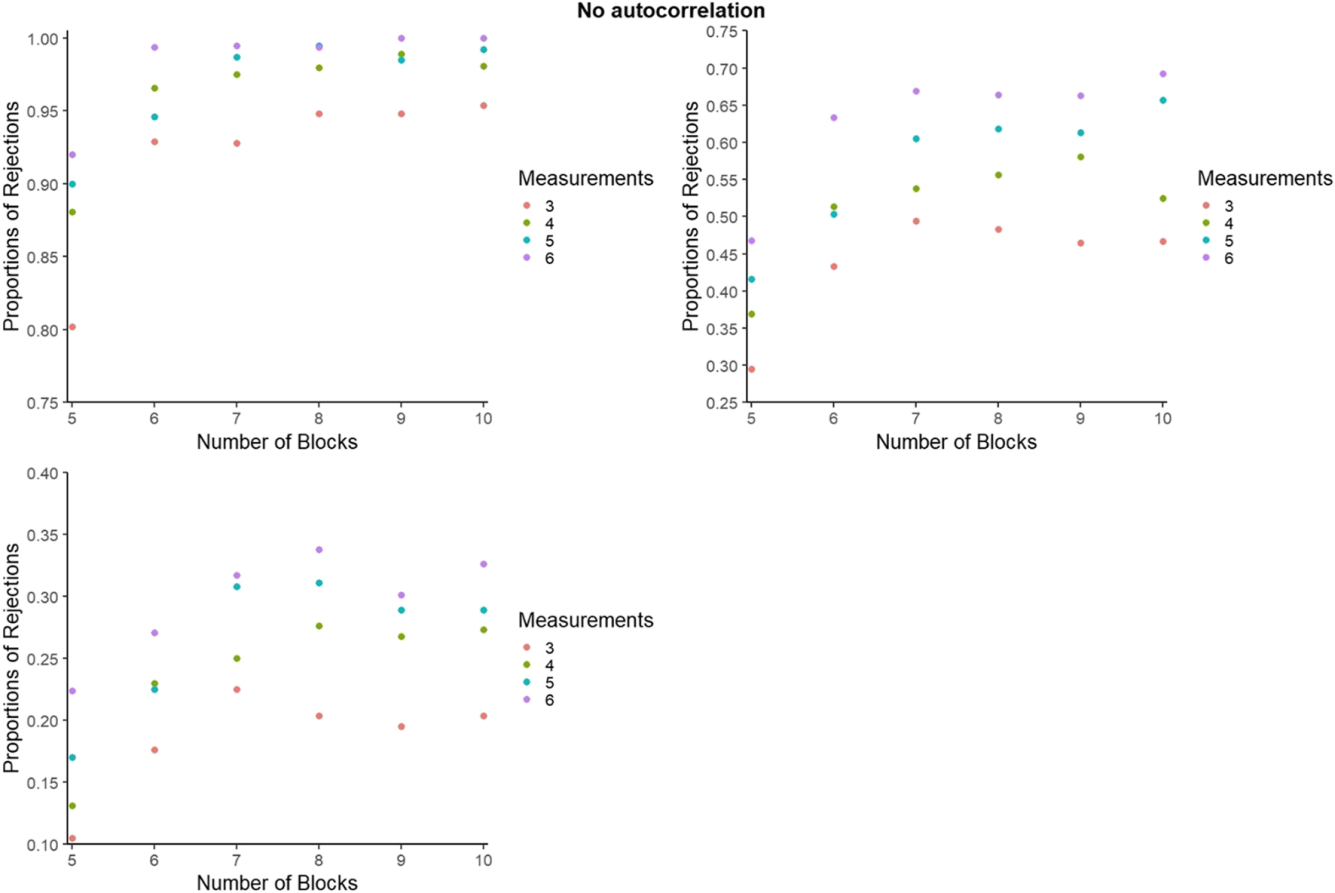
Statistical power estimates for blocked alternating conditions randomization, according to the number of blocks and the number of measurements per phase. Independent data. Random variability: upper left - 10%, upper right - 25%, bottom - 50%

**Fig. 12:**
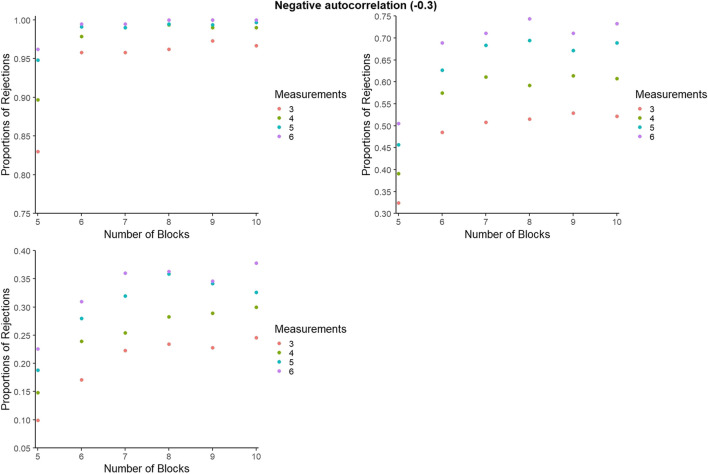
Statistical power estimates for blocked alternating conditions randomization, according to the number of blocks and the number of measurements per phase. Data with negative autocorrelation. Random variability: upper left - 10%, upper right - 25%, bottom - 50%

**Fig. 13:**
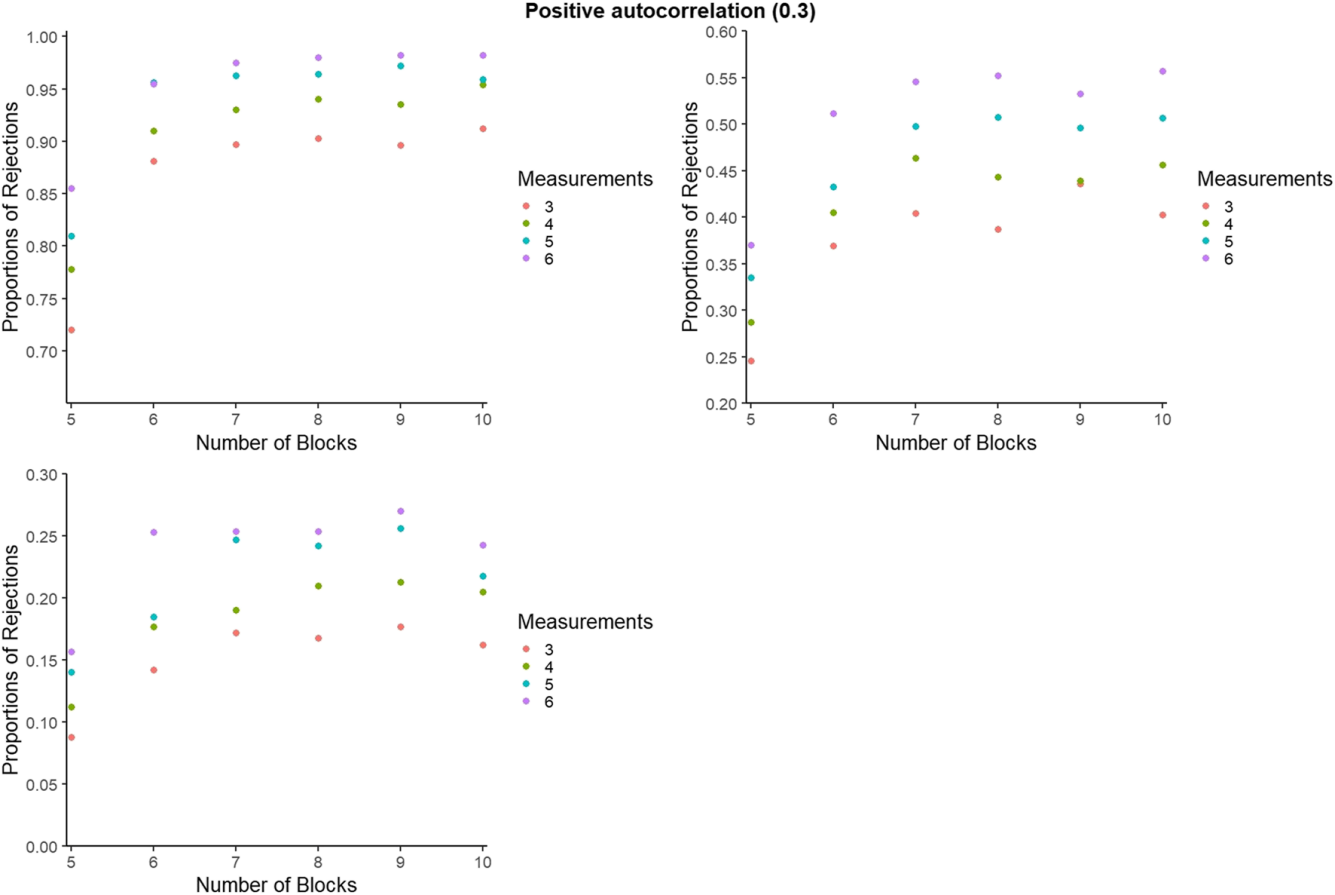
Statistical power estimates for blocked alternating conditions randomization, according to the number of phases and the number of measurements per phase. Data with positive autocorrelation. Random variability: upper left - 10%, upper right - 25%, bottom - 50%

For representing the statistical power for BAC, as for PCM, to avoid excessive cluttering and to better zoom in on the differences within and between the number of blocks and number of measurements per phase, the *y*-axes were set separately for each panel. However, figures with axes ranging from 0 to 1 can be consulted from the online supplementary material called Appendix C, available at https://osf.io/2jqtb/. Figures 11, 12, and 13 refer to independent data, negative autocorrelation, and positive autocorrelation, respectively.

In general, statistical power is lowest for positive autocorrelation for all simulation conditions, whereas power increases with the number of measurements per phase. The largest increase in power appears when increasing the number of blocks from five to six, and when increasing the number of data points per phase from three to four. The exception is positive autocorrelation. More specifically, statistical power decreases with a higher number of blocks (e.g., going from eight to nine blocks) for high variability (50%). In the presence of low variability (10%), power reaches 1 when there are at least six blocks with six measurements available. The exception is in the presence of positive autocorrelation, where power does not reach 1 for any of the conditions. In the presence of low variability (10%), statistical power is at least .80 for all simulation conditions and at least .90 for six blocks or more. The exception for both is positive autocorrelation.

## Discussion

### Comparison with previous findings

To the best of our knowledge, this is the first simulation study on any data analytical technique applicable to CCDs (including randomization tests). However, the current results can be related, with reservations, to previous findings. On the one hand, it is possible to compare the results of the current simulation with the results from a simulation study on a randomization test applied to a reversal ABAB design, given that the PCM randomization procedure is borrowed from reversal designs (Onghena, 1992). Specifically, Manolov and Solanas (2008) included conditions such as a series length of 20 measurements, four phases, and a minimal phase length of three, which is also present in the current study. They report a reduction of power for positive autocorrelation (as compared to negative or zero autocorrelation), which is also present in the current results. Power reaches .80 when the effect size is relatively large (a standardized mean difference of 2), and it is approximately .60 for a standardized mean difference of 1.4, whereas for PCM here we found power greater than .80 when the random variability is 10% of the criterion level and .60 when this variability is 25%.

Additionally, in relation to PCM, Manolov et al. (2010) studied conditions with a series length of 30 measurements, four phases, and a minimal phase length of five. They report that the presence of autocorrelation did not affect type I error rates. However, they also report that when the first and last phases are shorter (as in the triangular pattern studied here), positive autocorrelation is associated with a more liberal test. This finding is similar to our results of increased type I error rates for the triangular pattern of phase lengths, for series lengths of 24 and 28. We did not replicate this result for a series length of 30, but our conditions with such a series length included five or six (rather than four) phases and a minimal phase length of three (rather than five, as in Manolov et al., 2010). Regarding power, Manolov et al. (2010) report lower power when later phases are longer. We obtained similar results for designs with four phases (with a series length of 24 and 28), when comparing the increasing pattern with the uniform pattern.

Finally, in relation to PCM, Levin et al. (2012) report four separate but related investigations in their article. Their Investigation 4 included a design with six phases and 36 measurements in total, which is a condition also included in our study. A decrease of power is reported with the increase of positive autocorrelation, with power being approximately .80 for independent data for a standardized mean difference of 1. In our simulations, we obtained a similar estimate of power when the random variability was 25% of the criterion level.

On the other hand, it is also possible to compare the results of the current study with the results from a simulation study on a randomization test applied to alternating treatments design with block randomization (also called randomized block designs) (Onghena & Edgington, 2005), given that the randomization procedure is similar to BAC. Specifically, Levin et al. (2012) report several simulations studies, with their Investigations 1 and 2 including a “random pair” design with 24 and 12 measurements, respectively, organized in 12 or 6 blocks (i.e., there is a single measurement per condition each time that it occurs). They report an increase in power with higher positive autocorrelation, which is at odds with our results for the BAC procedure, but we only studied up to 10 blocks and at least three measurements per phase. The results in terms of controlling type I error rates are concordant.

Additionally, in relation to BAC, Manolov (2019) reports results on an alternating treatment design with block randomization, for series lengths between 10 and 24, with the number of blocks of pairs of conditions ranging from 5 to 12. As in Levin et al. (2012), the number of measurements each time that a condition occurs is one. In Manolov (2019), type I error rates were controlled, with no effect of autocorrelation, as reported in Table 3 here. For a standardized mean difference of 1, a power of .80 was not achieved, whereas for a standardized mean difference of 2, it was already present for as few as six blocks (12 measurements). Here, we observed power estimates reaching .80 for six blocks (36 measurements) when the random variability was 10% of the criterion level.

### Practical implications

The current recommendations highlight the results obtained for positively autocorrelated data, due to two reasons. On the one hand, positive serial dependence (i.e., greater similarity across measurements adjacent in time) is more likely to be expected on a logical basis (Jones et al., 1977; Sideridis & Greenwood, 1997), although a variety of levels has been found in reviews of SCED data (Barnard-Brak et al., 2021; Shadish & Sullivan, 2011; Solomon, 2014). On the other hand, considering the difficulty in estimating autocorrelation precisely in short series (Arnau & Bono, 2003; Huitema & McKean, 1991; Shadish et al., 2013), a conservative approach is warranted, taking into account the lower power observed here for conditions with positive serial dependence.

For positively autocorrelated data, for PCM, power is above .80 even for series as short as 15 measurements, when the random variability is not more than 10% of the criterion level, whereas 42 measurements are needed to reach this power when the random variability is 25%. For independent data, power is sufficient already with 28 measurements even when the random variability is 25%. Including a reversal to a previous criterion level for PCM leads both to controlling type I error rates and increasing power.

For BAC, for positively autocorrelated data and random variability not higher than 10% of the criterion level, statistical power is sufficient (at or above .80) when there are ten phases with five measurements each (i.e., for a total series length of 50). Additionally, power is above .70 when there are ten phases with three measurements each (i.e., for a total series length of 30), and power reaches .80 for a series length of 30 for independent data.

According to the results from our review (presented in Table 1), considering a median number of phases (five) and the median phase length (four) leads to a median series length of 20, whereas the corresponding means (6.14 and 5.81, respectively) lead to an average series length of more than 30. Thus, statistical power appears to be sufficient for typical CCD conditions only if the data are independent or the random variability is low (10%), in terms of the number of phases when the PCM procedure is followed and in terms of the total series length for both PCM (with a minimum phase length of three) and BAC (with a phase length of three for all phases). In case there is positive autocorrelation and random higher variability (25%), longer series are required: for instance, for PCM, the 42 measurements needed would represent percentile 75 of the reviewed CCD data (seven phases and six measurements per phase).

Based on the results of the current simulation study, several recommendations about the two procedures can be made for applied research. For PCM, we recommend including a reversal to a previous criterion level, as it had a positive effect both on type I error rates and statistical power. By comparing the results of simulation conditions with the same series length, it appears to be beneficial in terms of statistical power to have fewer phases (i.e., with the possibility of them being longer) rather than more phases. For BAC, power is larger when more measurements per phase are available, even when the number of phases is the same. For both PCM and BAC, the obvious recommendation of having as many measurements as possible can be made. If we wanted to compare PCM and BAC for the same number of measurements, in order to decide whether one of the randomization procedures is more powerful, the following can be found in the results of the simulation (https://osf.io/9hqjr). For 30 measurement occasions and an autocorrelation of .3, the statistical power for BAC (five blocks of two conditions or phases, three measurements per phase; 32 possible randomizations) is .72 for 10% random variability and .25 for 25% random variability, whereas the statistical power for PCM (five phases with a minimum phase length of three measurements; 3876 possible randomizations) is 1.00 and in the range .61–.65 for 10% and 25% random variability (for all three phase length patterns studied: uniform, increasing, and triangular). The PCM results for 30 measurements and six phases (6188 possible randomizations) are similar, although the power for 25% random variability is in the range .51–.55. Thus, for this specific number of measurements (which can be considered typical, as the mean number number of phases is 6.14 with an average phase length of 5.81, as per Table 1), power is greater for PCM. More nuanced results for different conditions are presented in the figures.

### Additional contribution: R code

R code for carrying out a randomization test for an individual CCD study is available at https://osf.io/dbfxk/. For both PCM and BAC, the first step is to load a data file. The required structure of the data file is illustrated in https://osf.io/dbfxk/. Four columns are necessary with the following corresponding column names: (1) “Phase” includes successive letters that mark the different phases; (2) “Session” includes the measurement occasion, ranging from 1 to *n*; (3) “Scores” includes the obtained measurements; and (4) “Criterion” includes the implemented criterion for each phase. For PCM, the user has to specify the minimal phase length, whereas for BAC, it is necessary to make sure that the number of experimental phases is even. Afterwards, the code is just copied and pasted into the R console. A *p*-value is obtained, as well as a graphical representation, similar to Figs. 4 and 5. For PCM, there is code for a Monte Carlo sample of all possible randomizations and two codes for using a systematic randomization distribution, as there are different ways in which a systematic list of all possible randomizations can be obtained. (The one marked by the letters PO is more efficient than the one marked by the letters RM.)

### Limitations and future research

Regarding the simulation study, an initial remark refers to the definition or specification of random variability when generating the data. Random variability was defined around the criterion level, given that the important thing in CCDs is the matching or close correspondence between the measurements and the criterion level (Kazdin, 1989; Ledford & Gast, 2018a) and not the size of the difference between the criterion levels. In contrast, the amount of difference between criterion levels is usually determined in such a way as to make progress feasible for the specific individual and behavior studied. This is why the random variability is not considered relative to the difference between criterion levels.

The results of the current study are necessarily limited to the conditions included in the simulation. Regarding BAC, the simulation included only blocks of two criteria and only phases of equal lengths, and future research could extend these conditions to varying phase lengths. Having different phase lengths for the intervention phases is both a methodological recommendation (Klein et al., 2017) and the likely result of the frequent use of a priori established mastery criteria for deciding when a criterion level has been sufficiently met (Manolov et al., 2020), However, even with this response-guided nature of determining the intervention phase length according to the emerging data pattern, the resulting intervention phases can have equal lengths (e.g., Shrestha et al., 2013). In any case, it should be noted that PCM entails determining phases lengths a priori (and phases may end up being of the same or different lengths), whereas BAC requires randomizing only the order within blocks and is applicable regardless of the exact length of the phases (equal or not).

Regarding PCM, not all possible combinations (patterns) of phase lengths were studied as representing the actual data. Future research can focus on data patterns additional to the uniform, increasing, and triangular patterns. Moreover, it is necessary to dig deeper into the excessively high type I error rates for triangular patterns in certain conditions (i.e., for some series lengths and number of phases, but not for all). In addition, future simulation studies could focus on response-guided PCM randomization tests for CCD.

A different line of future research could focus on a version of the CCD, called the distributed criterion design, including elements of multiple-baseline and reversal designs (McDougall, 2006). This version entails studying simultaneously the same behavior in different contexts or several related behaviors. A randomization procedure could be proposed and tested for this design as well.

Finally, the present text has not dealt with trend for several reasons, related both to the design and to the data analytical approach studied here. In general, CCDs are used for making progressive changes in the target behavior, and thus, trends can take place. However, if the design of the study is appropriate, a general trend should resemble a stepwise pattern (i.e., visually appearing like stairs), with relative stability around each step (level). Against the possibility of a general trend, it would be important to follow Klein et al.’s (2017) recommendations to vary phase magnitude (i.e., not all differences between criterion levels are the same) and/or implement a mini-reversal (i.e., go back to a less stringent criterion). This would help in checking whether the progression of the target behavior may be due to a general improving trend or is a function of the manipulation.

From a data analytical perspective, the test statistic can be expected to detect as unfavorable (i.e., against the idea that the measurements match the criterion levels) situations in which any trend may make the measurements obtained in one phase be closer to the criterion level of another phase. Thus, if any potential within- or between-phase trends would have fit the data better under any other randomization, then the randomization distribution (e.g., Fig. 4) will reflect this. Moreover, these quantifications (test statistic and randomization distribution) can be complemented with visual inspection to help in detecting whether there is a general trend, or a floor/ceiling effect not related to the criterion levels (i.e., the participant reaching the desired final level of the target behavior earlier than planned). In any case, more research is needed to understand the presence (or lack thereof) of trends in empirical CCDs.

## Conclusions

Randomization tests can be used for CCD data, using two different randomization procedures. Type I error rates are generally controlled, except under PCM when the phase-length pattern has a triangular shape. For the PCM randomization, statistical power is sufficient for series with at least 28 measurements if data are independent, which are common in published CCD research, but more measurements are necessary (i.e., 42) if researchers do not want to assume lack of autocorrelation. For BAC, more measurements are necessary for having sufficient power than for PCM. User-friendly R code is available for performing the randomization test using the two randomization procedures.

## Footnotes

1 It is assumed that the reader has a basic familiarity with single-case experimental methodology. Recommended readings for readers wishing to familiarize themselves with the methodology include textbooks by Barlow et al. (2009), Kazdin (2020); Ledford & Gast (2018b), as well as articles by Ledford et al. (2019), Plavnick & Ferreri (2013); Tanious & Onghena (2019).
